# Supplementary material for: Inflammatory factors and risk of lung adenocarcinoma: a Mendelian randomization study mediated by blood metabolites
Source: Front Endocrinol (Lausanne). 2024 Aug 27;15:1446863. doi: 10.3389/fendo.2024.1446863 (PMC11384989; doi:10.3389/fendo.2024.1446863)
Supplement: Supplementary file 1 [file DataSheet1.zip › supplementary files/S2/GCST90200725/sensitivity-analysis.pdf]

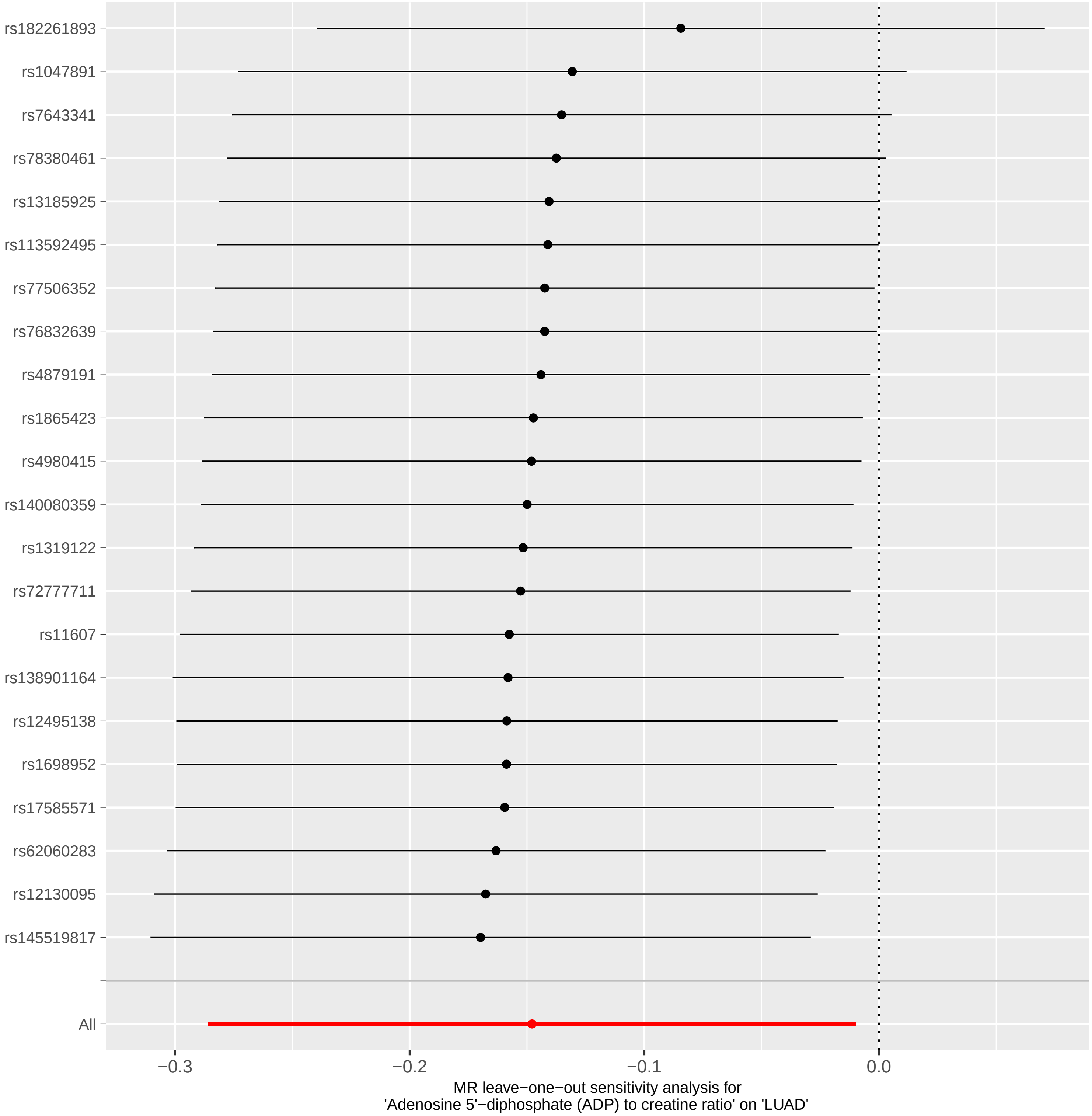
rs182261893
rs1047891
rs7643341
rs78380461
rs13185925
rs113592495
rs77506352
rs76832639
rs4879191
rs1865423
rs4980415
rs140080359
rs1319122
rs72777711
rs11607
rs138901164
rs12495138
rs1698952
rs17585571
rs62060283
rs12130095
rs145519817
All
−0.3
−0.2
−0.1
0.0
MR leave−one−out sensitivity analysis for
'Adenosine 5'−diphosphate (ADP) to creatine ratio' on 'LUAD'
